# Apply Graph Signal Processing on NILM: An Unsupervised Approach Featuring Power Sequences †

**DOI:** 10.3390/s23083939

**Published:** 2023-04-12

**Authors:** Bochao Zhao, Xuhao Li, Wenpeng Luan, Bo Liu

**Affiliations:** School of Electrical and Information Engineering, Tianjin University, Tianjin 300072, China

**Keywords:** non–intrusive load monitoring, graph signal processing, state transition sequences, dynamic time warping

## Abstract

As a low-cost demand-side management application, non-intrusive load monitoring (NILM) offers feedback on appliance-level electricity usage without extra sensors. NILM is defined as disaggregating loads only from aggregate power measurements through analytical tools. Although low-rate NILM tasks have been conducted by unsupervised approaches based on graph signal processing (GSP) concepts, enhancing feature selection can still contribute to performance improvement. Therefore, a novel unsupervised GSP-based NILM approach with power sequence feature (STS-UGSP) is proposed in this paper. First, state transition sequences (STS) are extracted from power readings and featured in clustering and matching, instead of power changes and steady-state power sequences featured in other GSP-based NILM works. When generating graph in clustering, dynamic time warping distances between STSs are calculated for similarity quantification. After clustering, a forward-backward power STS matching algorithm is proposed for searching each STS pair of an operational cycle, utilizing both power and time information. Finally, load disaggregation results are obtained based on STS clustering and matching results. STS-UGSP is validated on three publicly accessible datasets from various regions, generally outperforming four benchmarks in two evaluation metrics. Besides, STS-UGSP estimates closer energy consumption of appliances to the ground truth than benchmarks.

## 1. Introduction

Demand-side management (DSM) is an effective tool to balance electricity supply and demand [1]. As a popular DSM application, load monitoring can be carried out intrusively or non-intrusively. Intrusive load monitoring (ILM) requires extra sensor installation at plug-ends, while non-intrusive load monitoring (NILM) does not. NILM technique aims to disaggregate power consumed by each appliance and identify their operational states by only one sensor in mains, via analysing aggregate power readings using software tools. The concept of NILM was raised by G. W. Hart in 1980s [2], as a low-cost and user-friendly alternative to load monitoring sensors. By offering fine-grained electricity consumption feedback, including the categories, operational power ranges and usage habits of major appliances, NILM enriches smart metering benefits and supports DSM [3].

According to the sampling rates of power readings to be disaggregated, NILM tasks can be classified as high-rate (above 1 Hz, usually in kHz and MHz) and low-rate (from 1/60 Hz to 1 Hz) [4]. Although high-rate measurements carry more detailed information (e.g., appliance transition state), the cost of data collection and storage is high. Comparing to high-rate NILM, NILM methods for low-rate measurements perform competitively in some scenarios, which are available to commercial meters deployed worldwide for billing purposes. Therefore, low-rate NILM approaches are regarded as feasible solutions, capturing more attention recently. So far low-rate NILM approaches have been developed based on the hidden Markov model (HMM) and its variants [5], random forest (RF) [6], decision tree (DT) [7], neural networks [8,9,10,11], support vector machines (SVM) [12], *k*-means [12], fuzzy c-means (FCM) [13], mathematical programming [14,15,16] and graph signal processing (GSP) [17,18], etc. Such NILM approaches can be supervised or unsupervised, depending on whether sub-metering data or appliance usage labelling is required for training. That is, for making supervised NILM approaches work, either plug-end sensors or users survey is required [5,8]. For more practical scenarios where neither sub-metering nor labelling is available, unsupervised methods can still work [19]. Therefore, unsupervised NILM methods have the potential to disaggregate loads from the mains of real ‘unseen’ households.

HMM is widely used in pattern recognition and shows its superiority in identifying loads with strong periodicities. However, HMM suffers from the exponential increase in computational cost when more appliances are added. For improving disaggregation performance and efficiency, sparse Viterbi algorithm is proposed in [5] for efficiently computing sparse matrices in power disaggregation task. Factorial hidden Markov model (FHMM), as an HMM extension, can be utilized to identify multi-state appliances. In [20], each appliance is modelled by a bivariate HMM, where emitted symbols are joint active-reactive power signals. A hybrid algorithm is proposed in [21], where a two-state FHMM is used to decrease computational complexity, with subsequence dynamic time warping for performance improvement. In [22], two multi-observation FHMM variants are proposed, showing extra reactive power observation and state duration distribution feature help improve performance in industrial scenarios. A modified FHMM is applied to NILM in [23], where dependency models for operational states of all appliances can be built based on electricity profile variation. However, over-estimation may occur in the above-mentioned HMM-based NILM methods when facing non-periodic loads.

As deep learning (DL) becomes popular in recent years, neural networks with various architectures have been applied to solve the NILM problem, such as convolutional neural network (CNN), long short-term memory (LSTM), stacked denoising autoencoder (DAE) and sequence-to-point (seq2point). In [8], CNN, LSTM and stacked DAE are applied to NILM, where CNN and DAE outperform LSTM due to their wider range of trainable parameters. However, for frequently used appliances, the performance of CNN and DAE drops. A seq2point CNN framework is proposed in [10], generally outperforming sequence-to-sequence networks in load disaggregation tasks. To avoid sub-metering for the target houses, transfer learning across houses and datasets is studied in [11]. However, its performance relies on the similarity in power usage between the source dataset for training and the data collected from target houses. Moreover, sub-metering for the target houses is also required for network fine-tuning. For tackling this problem, a self-supervised NILM approach based on seq2point CNN is proposed in [9], consisting of a unsupervised pre-text task and a network fine-tuning task. However, as the pre-text task is heuristically set, explaining its impact on disaggregation performance is hard. Note that the implementation of most DL-based NILM approaches on commercial end-devices weight calculation and storage hinder their implementation on commercial end-devices. Therefore, A light-weight and scalable NILM approach is proposed in [24] to break such limitations, where transient sequences are segmented based on detected turn-ON events. Then a hybrid of CNN and *k*-NN is used to identify loads. However, high-rate measurements sampled at 100 Hz are required for power sequence extraction. A MobileNet is proposed in [25], where TensorFlow Lite is employed on a light-weight architecture for further compression, thus reducing memory and training period requirement. However, performance drop is observable caused by further compression.

Machine learning has been applied to identify and disaggregate loads, including SVM [12,26], *k*-NN [27], DT [7], RF [6], *k*-means[12], FCM[13], mean-shift [28], etc. In [26], load state transition events are detected heuristically and classified by an SVM model, featuring duration, average power, maximum power and power variance. A low-complexity hybrid NILM approach is proposed in [12], where *k*-means is utilized to refine the training dataset for the following SVM classifier. The results show the *k*-means-based training data refinement contributes to both disaggregation accuracy and efficiency. A NILM framework is proposed in [27], with a local power histogramming descriptor for feature extraction and an improved *k*-NN architecture for classification. Ref. [7] identifies loads by a DT-based classifier, with dynamic time warping (DTW) distance representing similarities between extracted state transition events. In [6], the Fourier series transformed from the aggregate and weather information are featured in a RF classifier. However, more costs are required for extra sensors. In addition to such supervised NILM methods, unsupervised ones have also been investigated, e.g., the NILM approaches proposed in [12] and [13] are unsupervised, achieving competitive performance. However, prior knowledge including the number of clusters is required. For breaking this limitation, Liu et al. apply mean-shift algorithm to group the transient states detected based on their magnitudes [28]. Note that small bandwidth is set for guaranteeing NILM performance while increasing convergence duration.

Unlike NILM approaches based on machine learning with multiple disaggregation tasks for each appliance, optimization-based ones can disaggregate all target loads via a single task [16]. In [2], combinatorial optimization (CO) is applied to NILM by searching the optimal combination of appliances’ operational states with total power closest to the aggregate via heuristic algorithms like genetic algorithm. However, CO is sensitive to unknown loads, leading to over-estimation. To solve this problem, integer programming (IP) approaches can be utilized. Bhotto et al. propose a load disaggregation approach based on aided linear IP in [14], with improvement on feature extraction, constraint selection, pre- and post-processing. However, when disaggregating a large number of appliances, global optimization becomes hard. A two-stage NILM scheme is proposed in [15], where operational state extraction is followed by load disaggregation via a hybrid of mixed-integer IP and penalized correction matrix. However, its performance is easily affected by noises. In [16], mixed-integer optimization is utilized to model appliance operational states, and then IP is used to solve energy disaggregation problem with additional penalty terms and constraints. However, specialized solvers are required for the measurements collected per second due to efficiency concern.

GSP, as an emerging signal processing tool to represent stochastic properties of signals using graphs, has also been employed in the NILM task. In [29], a supervised GSP-based NILM method is proposed, where the graph total variation is minimized, referring to generally piece-wise smoothness of the underlying graph signal. For performance refinement, simulated annealing is applied as post-processing to minimize the difference between the aggregate and the sum of disaggregated power in [30]. Note that active power change events are featured in training in both [29] and [30]. To perform GSP-based NILM in an unsupervised manner, GSP concepts are utilized multiple times in choosing adaptive thresholds, clustering, and switching events matching in [17]. Since only power change events are detected and featured, this method can only disaggregate loads with close power magnitudes of their switching ON and OFF events. Besides, transients lasting longer than the sampling period may be segmented as consecutive smaller events, ruining matching procedure. Both drawbacks constrain robustness. Although pre-processing proposed in [31] can sharpen state transition edges in power signals for performance improvement, transient information is vanished. Instead of widely-used power change events, steady-state sequences are segmented and featured by a GSP-based NILM method in [18]. Since the extracted sequences may differ in length, DTW is utilized for similarity calculation. However, its performance is sensitive to the simultaneous operation of multiple loads and measurement noises.

Driven by the aforementioned shortcomings of current NILM works, an unsupervised GSP-based NILM approach, STS-UGSP, is proposed in this paper, featuring sequences adaptively segmented from the aggregate power signals. Alternative to the steady-state sequence defined in [18], state transition sequence (STS) is defined in this paper, consisting of ‘rising’ and ‘falling’ power sequences. A ‘rising’ sequence refers to the starting power transient of an operational state, while its ending power transient can be represented by a ‘falling’ sequence. In this paper, DTW is utilized in graph edge weighting to calculate distances among extracted STSs for similarity measure as in [18]. Two classic NILM methods, are selected as baselines, as CO implemented in publicly available NILMTK toolbox [32] and sparse HMM (SHMM) proposed in [5]. For a more representative comparison, two GSP-based NILM solutions are also benchmarked, which are proposed in [17] and [18], respectively. The validation is carried out on three publicly available datasets, AMPds dataset[33], REFIT dataset [34] and REDD dataset [35]. Our main contributions can be summarised as:

- an improved STS extraction method is proposed for capturing sequences containing each complete switching event between operational states of an appliance;
- in the proposed unsupervised GSP-based NILM approach, each graph node maps a STS, where the adjacency matrix is weighted based on DTW distances between STSs;
- a forward-backward power STS matching algorithm is proposed for matching each ‘rising’ STS to the optimal ‘falling’ one, based on their power ranges and time interval;
- the proposed method is validated on open-access AMPds, REFIT and REDD datasets, benchmarked with state-of-the-art GSP-based NILM methods using different features and two classic NILM approaches based on SHMM and CO.

The rest of this paper is organised as follows. In Section 2, we introduce the preliminary knowledge of DTW and GSP. The details of the proposed method are explained in Section 3. In Section 4, we clarify the experimental setup and evaluation metrics. The whole experimental results are demonstrated and discussed in Section 5. Section 6 consists of a brief conclusion and future work.

## 2. Preliminary Knowledge

### 2.1. Dynamic Time Warping

Traditionally, time series similarity is measured by either lock-step measures or elastic measures. Lock-step measures, such as well-known Euclidean distance, conducting one-to-one comparisons, are applicable only for the time series with the same length. Alternatively, DTW, as an elasticity measure allowing one-to-many comparisons, enables similarity measuring of time series with unequal lengths.

Since proposed in [36], DTW has been applied to NILM problem to calculate the similarity between sequences extracted from measurements [37] (e.g., active power, reactive power, etc.). Given two sequences of arbitrary lengths *m* and *n* as $p=[p_{1},\ldots ,p_{m}]$ and $q=[q_{1},\ldots ,q_{n}]$, DTW distance aims to find minimal mapping paths between p and q. D represents cost matrix and the element D(i,j) is the accumulated DTW distance between points $p_{1}$ to $p_{m}$ and points $q_{1}$ to $q_{n}$, and $D_{m,n}$ is the final distance between the two vectors. The DTW distance calculation for p and q is shown in Algorithm 1.
**Algorithm 1:** DTW distance calculation
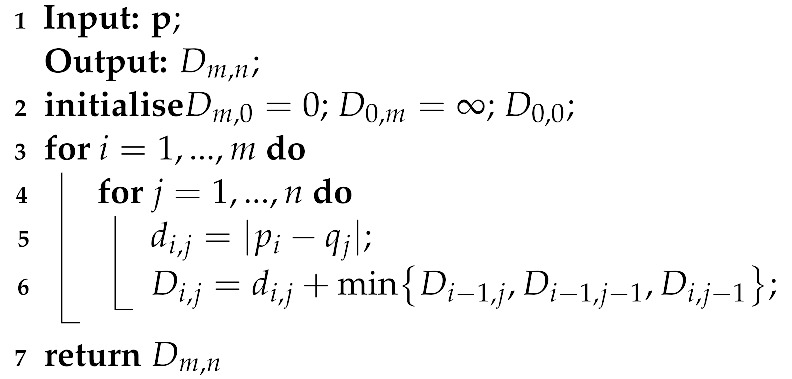


**Figure 1:**
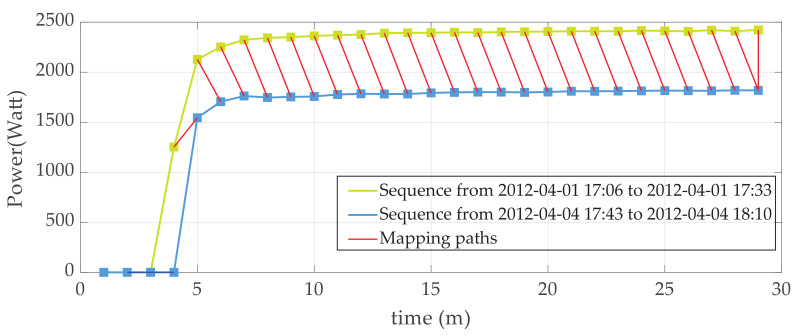
Demonstrative mapping paths in DTW distance calculation for two typical power sequences.

An example of DTW distance calculation between ‘rising’ power sequences extracted from AMPds dataset is illustrated in Figure 1.

In Figure 1, the optimal mapping paths between power sequences are shown in red.

### 2.2. Graph Signal Processing

Let $X=\left\{x_{1},x_{2},\ldots ,x_{N}\right\}$ denote a *N*-length set, where $x_{i}$ can be a vector or set. Then, an undirect graph for X can be built as G=(V,A), where $V=\left\{v_{1},v_{2},\ldots ,v_{N}\right\}$ is a set of vertices on the graph, with each $x_{i}$ corresponding to $v_{i}$. The graph adjacent matrix A is a N×N symmetric matrix and each entry $A_{i,j}$ represents the weight of edge between graph nodes $v_{i}$ and $v_{j}$, representing the similarity or correlation of $x_{i}$ and $x_{j}$. The value of $A_{i,j}$ is usually defined by Gaussian kernel function as in [38]:

(1) $$A_{i,j}=\exp\left\{-\frac{{\left|dist(x_{i},x_{j})\right|}^{2}}{\rho^{2}}\right\},$$

where ρ is a scaling factor, and $dist(x_{i},x_{j})$ denotes the distance between $x_{i}$ and $x_{j}$, which can be calculated as Euclidean distance or DTW distance.

Graph signal s is a mapping from V into real domain R, thus, a relationship can be built as $s_{i}=f\left(v_{i}\right)$. Note that if a graph signal is piece-wise smooth, its global smoothness is generally small. Then, the signal global smoothness can be formulated as [39]:

(2) $$S_{p}\left(s\right)=\frac{1}{p}\sum_{i\in V}{\left[\sum_{j\in N_{i}}A_{i,j}{\left(s_{j}-s_{i}\right)}^{2}\right]}^{\frac{p}{2}},$$

where $N_{i}\in V$ is the set of vertices connected with $v_{i}$. Let D be a diagonal matrix, defined by $D_{i,i}=\sum_{j}A_{i,j}$, for i=1,…,N, then L=D−A denotes the graph Laplacian matrix. By setting p=2, a graph Laplacian quadratic form can be obtained via Equation (3) as [31]:

(3) $$S_{2}\left(s\right)=\frac{1}{2}\sum_{i,j}A_{i,j}{\left(s_{j}-s_{i}\right)}^{2}=s^{T}Ls.$$

Since $s^{T}Ls$ is generally small when graph signal s is piece-wise smooth, by solving optimization problem $\arg\min_{s}{∥s^{T}Ls∥}_{2}^{2}$, a closed form solution can be obtained as:

(4) $$s^{*}=L_{2:N,2:N}^{\#}\left(-s_{1}\right)L_{1,2:N}^{T},$$

where (.)${}^{\#}$ denotes the pseudo-inverse matrix.

## 3. Methodology

The proposed STS-UGSP consists of three stages: adaptive STS extraction, GSP-based STS clustering and forward-backward STS matching.

### 3.1. State Transition Sequence Extraction

Initially, an adaptive method for extracting STSs is proposed. Although a signal segmentation algorithm has been proposed in [18] for capturing steady-state sequences, the method proposed in this paper is different. We focus on the power sequences containing complete transients referring to operational states start or end, which are extracted for further clustering and matching. The proposed state transition sequence extraction algorithm is presented in Algorithm 2.

In Algorithm 2, for an *N*-length aggregate power signal p, two parameters $t_{0}$ and $t_{1}$ are heuristically set for constraining sequence length. *T* denotes a threshold for distinguishing power changes due to state transition and measurement noise, and γ is a preset factor. After initialisation, each power variation sample $\Delta p_{i}$ (calculated in Line 3) with magnitude no less than *T* and its previously neighbouring samples are assigned to a sequence e, based on the adaptive rules as Lines 5–12. Otherwise, $p_{i}$ is stored in e, as shown in Lines 13–17. Since a single ‘rising’ or ‘falling’ STS may contain multiple time-wise close state transitions, especially for the measurements with low granularity, further segmentation are designed. In Line 21, the *j*-th element $e_{i,j}$ is assigned to s as a container for storing sub-sequences. Then the indices of the maximum or minimum in each sub-sequence, which is segmented by judging consecutive samples above or below ${\bar{e}}_{i}$, are stored in l. The STSs satisfying heuristically set conditions are further segmented. Finally, all STSs are labelled. Note that for the cases with granularity reduced to 10 min and longer, the target to be disaggregated tends to be electricity profiles. Thus, state transition detection or STS extraction is no longer available.
**Algorithm 2:** STS extraction algorithm
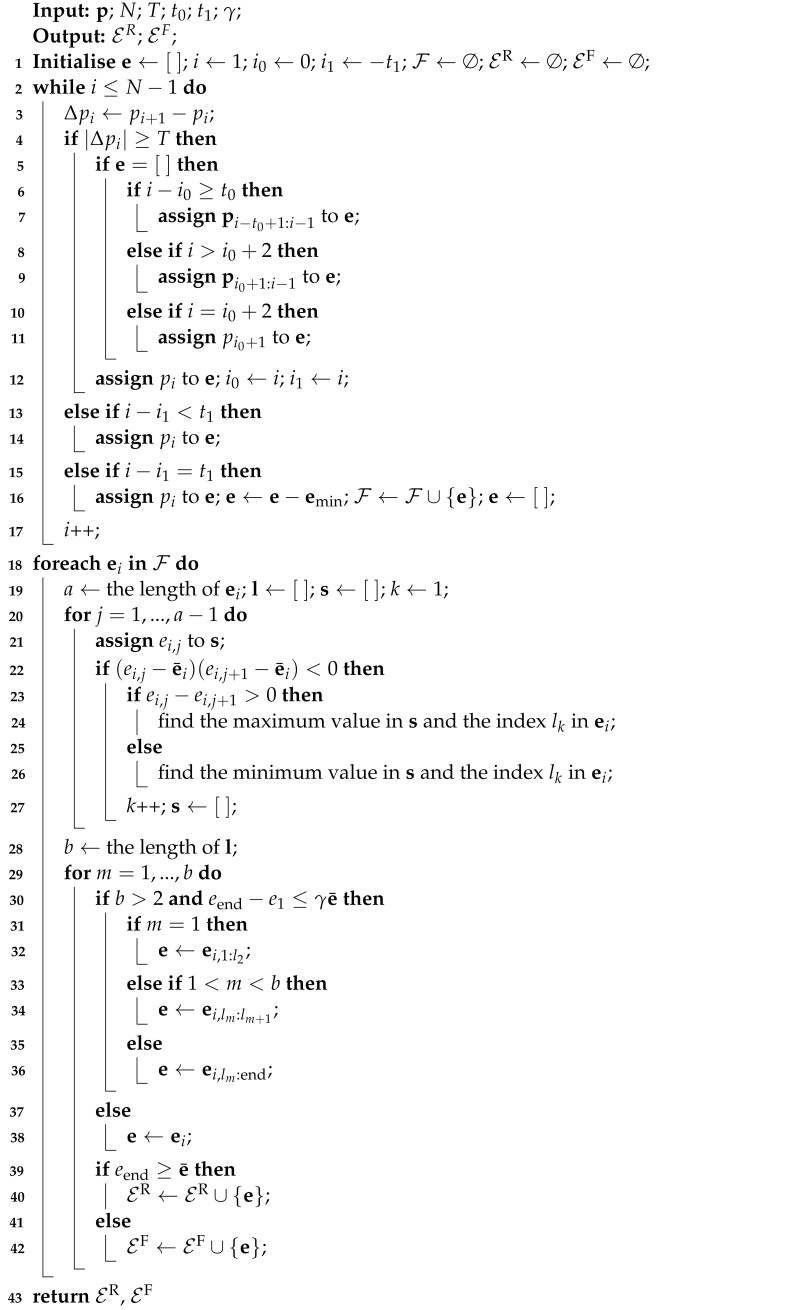


### 3.2. GSP-Based Power Sequence Clustering

**Figure 2:**
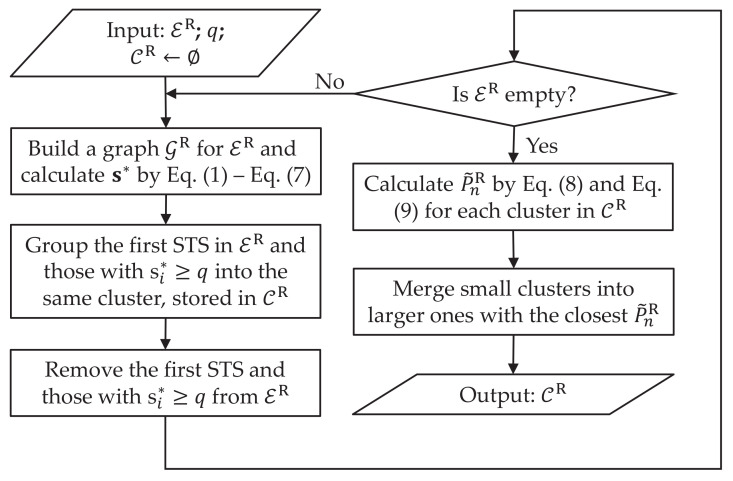
Flow chart of GSP-based STS clustering, where (.)* denotes the optimized signal.

Then GSP-based clustering is carried out for the extracted STSs. Note that the clustering steps for $E^{R}$ and $E^{F}$ are the same and independent, based on two graphs separately built for them. Therefore, taking clustering for $E^{R}$ as an example, its flow chart is shown as in Figure 2, the same as in [40].

Given a set $E^{R}$ containing *N* sequences, its graph is defined as $G^{R}$. In $G^{R}$, each node $v_{i}$ corresponds to a sample $s_{i}$ in graph signal s, as a mapping of sequence $e_{i}$ in $E^{R}$, for i=1,…,N. Note that the sequences in $E^{R}$ may differ in length, DTW distance is an appropriate tool to similarity measure. Then, let each entry $B_{i,j}$ in B represent DTW distance between sequences $e_{i}$ and $e_{j}$ in $E^{R}$, defined as:

(5) $$B_{i,j}=DTWdist\left(e_{i},e_{j}\right),$$

Since B is diagonal symmetric, its normalization can be conducted for each entry $B_{i,j}$ by:

(6) $$B_{i,j}=B_{i,j}/\mathrm{median}\left(B_{i,1:N}\right);$$

where median(.) represents the median value. Thus, the adjacency matrix can be defined as:

(7) $$A_{i,j}=\exp\left(-B_{i,j}^{2}/\rho^{2}\right).$$

Since in GSP-based clustering, the clustering target corresponding to $s_{1}\leftarrow 1$ can be randomly picked from the input [17], we pick the first STS as the target in this paper. Therefore, the remaining STSs are to be clustered, with corresponding samples in s initialised as zeros. Then, the optimized graph signal $s^{*}$ can be calculated by Equations (2)–(4), Since each $s_{j}$ corresponds to a sequence to be clustered, a fixed threshold *q* is used to select candidates. Thus, we group the first STS (with $s_{1}=1$) and those with $s_{j}^{*}\geq q$ into the same cluster, stored in $C^{R}$ as a sub-set. After removing such clustered STSs from $E^{R}$, the above-mentioned process is repeated for the updated $E^{R}$ until no STS is left. Like in [17], small clusters are merged with larger ones for simplifying further cluster matching. Assuming that the *n*-th cluster $c^{R,n}$ in $C^{R}$ contains *J* STSs, thus for selecting mergeable clusters, we define the *magnitude* of $c^{R,n}$ and each $c^{R,n,j}$ in it as Equations (8) and (9):

(8) $${\tilde{P}}^{R,n,j}=\left|c_{\mathrm{end}}^{R,n,j}-c_{1}^{R,n,j}\right|,$$

(9) $${\tilde{P}}^{R,n}=\frac{1}{J}\sum_{j=1}^{J}{\tilde{P}}_{n,j}^{R},$$

where $c_{1}^{R,n,j}$ and $c_{\mathrm{end}}^{R,n,j}$ denote the first and last elements in the *j*-th sequence of $c^{R,n}$. Then, the time instance of the largest power increase in $c^{R,n,j}$ is called *operation time* and represented by $t^{R,n,j}$, which is the *j*-th element of $t^{R,n}$. Then, each small cluster in $C^{R}$ with $J\leq J_{0}$ is merged to the larger one with the closest *magnitude*. Eventually, a set $C^{R}$ is obtained as the clustering result of ‘rising’ STSs. Similarly, by performing such clustering on the extracted ‘falling’ STS set $E^{F}$, $C^{F}$ can be obtained.

### 3.3. Forward-Backward Power STS Matching

In order to match the clusters of ‘rising’ STSs with those of ‘falling’ STSs, a forward-backward power STS matching algorithm is proposed, calling a sub-algorithm for pairing each STS from a ‘rising’ cluster to one in a ‘falling’ cluster, as in Algorithm 3.

In Algorithm 3, α and β are two heuristically set trade-off factors, where α+β=1, for balancing the magnitude of two STSs and their time intervals; τ is a general constraint of maximum operation time for each appliance; $t^{R,n}$ and $t^{F,m}$ are corresponding *operation time* of the *n*-th ‘rising’ cluster $c^{R,n}$ and *m*-th ‘falling’ cluster $c^{F,m}$. The function of Algorithm 3 is, given an arbitrary ‘falling’ STS in cluster $c^{F,m}$, to find its pair from candidates in a ‘rising’ STS cluster. One pairing criterion is that the time interval between two paired STSs is within τ. The other is that they are both close in magnitude and operationtime via computing $d_{i}$, where the minimum $d_{i}$ is stored in v. The proposed forward-backward power STS matching algorithm is shown in Algorithm 4 calling Algorithm 3 for pairing STSs from clusters. After initialisation in Line 2, ‘rising’ and ‘falling’ clusters are sorted in Line 4 based on their *magnitude* calculated in Line 3. Then forward steps are to look for all ‘rising’ STSs with paired ‘falling’ STSs based on Algorithm 3. After calling Algorithm 3, the minimum $\bar{v}$ is stored as *f* with corresponding index *m* stored as *b*. Then, all paired STSs detected by Algorithm 3 are stored in $R^{R,m}$ and $R^{F,n}$ and removed from original clusters. Otherwise, we break the loops. Lines 6–16 are repeated for the next ‘rising’ STS cluster until they are all traversed once. After forward steps, similar backward steps are conducted in Lines 17–27, where ‘rising’ clusters and ‘falling’ ones are swapped. Namely, backward steps act as looking for all ‘falling’ STSs with paired ‘rising’ STSs. Finally, all matched ‘rising’ and ‘falling’ STS pairs stored in $R^{R}$ and $R^{F}$ are obtained.
**Algorithm 3:** STS pairing algorithm
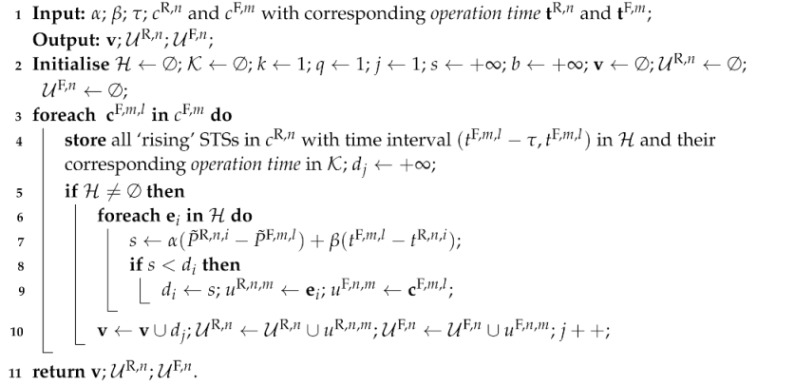


**Algorithm 4:** Forward-backward power STS matching algorithm

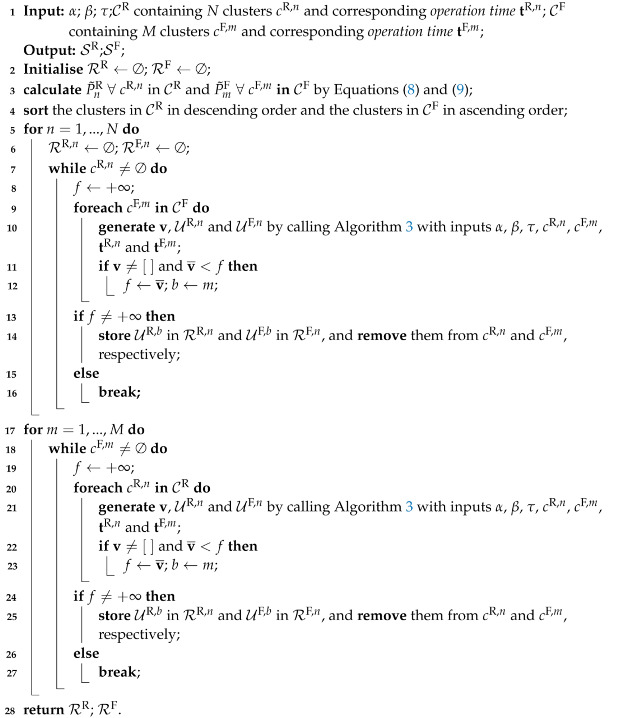



## 4. Experiment Setup

### 4.1. Dataset

To verify the effectiveness of the proposed method, a comparison against four benchmarks is conducted based on both three publicly accessible datasets, containing power data collected from real-world households. AMPds [33] dataset is collected from Canada and sampled at 1min resolution, REFIT [34] dataset is from the U.K., sampled at 8 s resolution and REDD [35] dataset is from U.S. houses at 1Hz resolution. Widely-used REDD dataset has neither fluctuant loads nor those with long-transient states, and its unknown loads are fewer than the other two datasets. AMPds dataset contains more multi-state appliances and various load types [33]. REFIT dataset is more challenging, due to non-negligible unknown appliances and measurement noise.

The loads to be disaggregated are abbreviated as follows: BP&L for basement plugs and lights; WD for washer dryer/clothes dryer; WM for washing machine/clothes washer; DW for dishwasher; F for fridge; HP for heat pump; WO for wall oven; B for bathroom GFI; KO for kitchen outlet; M for microwave; O for oven; S for stove; FFZ for fridge-freezer; K for kettle; T for toaster and FZ for freezer.

### 4.2. Parameter Selection

The parameter settings in the experiments are shown in Table 1.

The threshold *T* is set as 50 W for detecting both power changes and STSs in various methods. Since STSs extracted from different datasets differ in length due to inequivalent sampling rates, $t_{0}$, $t_{1}$ and τ are set heuristically. γ is set 0.2 for all datasets to distinguish the starting spike or multi-state. Comparing to *operation time* for the appliances in AMPds and REDD datasets, those for REFIT datasets are more distinguishable. Thus, a higher α is set for REFIT to facilitate feature representation. Since the adjacent matrix has been normalized, scaling factor ρ is set to 0.01 for all datasets. In GSP-based clustering, both $J_{0}$ and *q* are set to reduce falsely clustered STSs as in [40].

### 4.3. Evaluation Metrics

A metric is introduced for evaluating the proportion of unknown loads of datasets, followed by two metrics for NILM performance.

#### 4.3.1. Percent-Noisy Measure

Percent-noisy measure (%-NM) is a metric to quantify unknown loads in experimental datasets, defined as:

(10) $$\%-NM=\frac{\sum_{j=1}^{N}\left|p_{j}-\sum_{i=1}^{n}P_{j}^{i}\right|}{\sum_{j=1}^{N}p_{j}},$$

where $P_{j}^{i}$ is the actual power consumed at time *j* by device *i*. *N* is the signal length and *n* is the number of known appliances.

#### 4.3.2. *F-Measure*

*Precision* (PR), *Recall* (RE) and *F-measure* ($F_{m}$) are adopted as metrics to evaluate NILM performance and defined as in [23,31]:

(11) PR=TP/(TP+FP),

(12) RE=TP/(TP+FN),

(13) $$F_{m}=2\cdot TP\cdot FP/\left(TP+FP\right).$$

TP is the number of correctly identified events. FP is the number of events wrongly identified as the target. FN is the number of target events which are misidentified. Thus, for an arbitrary appliance, PR is the identification accuracy in all identified events, while RE is that in all actual events. $F_{m}$ is the harmonic mean of PR and RE, reflecting the correct identification level for per operational state per appliance.

#### 4.3.3. Disaggregation Accuracy

Besides, we use disaggregation accuracy Acc. as in [41] to evaluate how close the power estimation for an appliance is to what it is actually consumed. Acc. is defined as:

(14) $$Acc.=1-\frac{\sum_{j=1}^{N}\left|{\hat{P}}_{j}^{i}-P_{j}^{i}\right|}{2\sum_{j=1}^{N}P_{j}^{i}},$$

where ${\hat{P}}_{j}^{i}$ is the estimated power consumed at time *j* by device *i*. The factor of 2 is added in the denominator due to absolute values ‘double count’ errors. Noted that an Acc. close to 1 means accurate power estimation comparing to ground truth, while the one far away from 1 means poor performance.

## 5. Experimental Results and Discussion

In this section, experiments are conducted for the proposed STS-UGSP and four benchmarks under fair conditions as clarified before. For simplification, the benchmarks are represented as SS-UGSP [18], UGSP [17], SHMM [5] and CO [32].

### 5.1. NILM Results on AMPds Dataset

**Figure 3:**
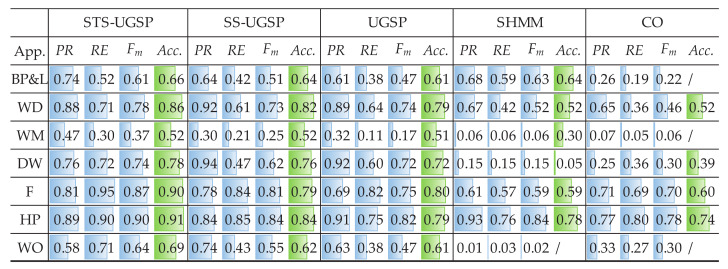
NILM Performance comparison on AMPds dataset.

Both $F_{m}$ and Acc. results are demonstrated as in Figure 3 for reflecting identification and disaggregation performance, separately. Note that for better visualization, colour bars are embedded in the blanks according to the results, i.e., the fully coloured blank means scoring 1 in its metric.

Overall, GSP-based NILM approaches outperform the baseline methods based on SHMM and CO, which is as expected and in line with the results shown in [31].

Comparing to SS-UGSP and UGSP, STS-UGSP performs better overall $F_{m}$, reaching 0.84. Take DW as an example, it takes 2 to 3 min from being turned ON to steady operation. Since such switching-ON transients last longer than the sampling period (1 min), multiple incomplete state transition events with lower power changes are detected. Thus, it’s hard to correctly pair such broken turn-ON events of DW to the corresponding turn-OFF ones based on power changes as in UGSP. Even worse, such events may be misidentified as other low-power loads, confusing event pairing. Furthermore, the steady-state events of DW are usually short and difficult to be captured in SS-UGSP. However, in STS-UGSP, extracted STSs contain the whole state transition processes. That is, the power transient characteristics of DW can be fully utilized when matching STSs. The other note-worthy appliance is F, which operates around 110 W, close to the power of low operational states of unknown multi-state appliances. Thus, high FP leads to low $F_{m}$ results for UGSP. However, STS-UGSP features DTW distance between STSs, which is more useful for solving the NILM problem, leading to improvement by 0.12 in $F_{m}$ and 0.1 in Acc. SHMM performs competitively in disaggregating HP with periodic operational cycles comparing to GSP-based methods, which is a general advantage of HMM-based approaches. Besides, SHMM slightly outperforms GSP-based methods in identifying multiple states of BP&L, as event matching is a complex task for the NILM approaches featuring power changes. Close results to SHMM can be observed for CO.

**Figure 4:**
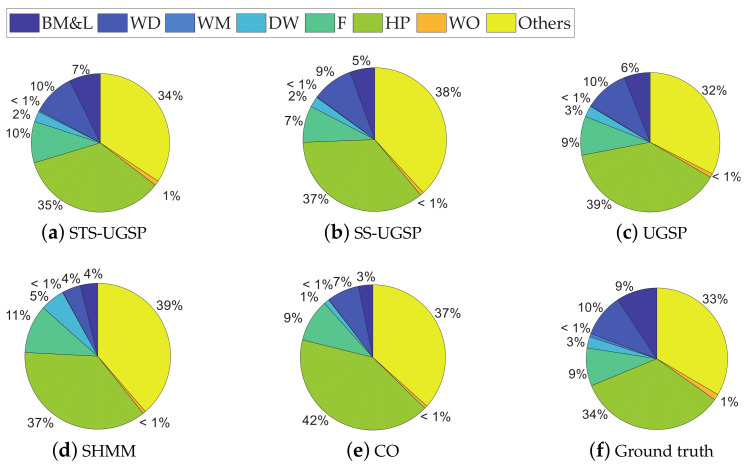
Pie charts of disaggregated energy shares on AMPds dataset.

In addition to $F_{m}$ and Acc. results, pie charts are drawn for illustrating energy shares disaggregated by various methods as in Figure 4. From Figure 4, STS-UGSP performs better than benchmarks for most appliances, consistent with the results shown in Figure 3, owing to HP which is the most power consumption appliance. It can be observed that HP has high disaggregation accuracy with STS-UGSP for its unique transient state. That is, HP can have high $F_{m}$ in DTW-based transient state clustering process and Acc. in matching step. From Figure 4, BP&L seems to be the most challenging load for energy estimation. Its long operation duration, usually lasting for 2 to 3 h, confuses matching in both STS-UGSP and UGSP. On the other hand, its large power fluctuations while operation makes the disaggregation task more complex.

**Figure 5:**
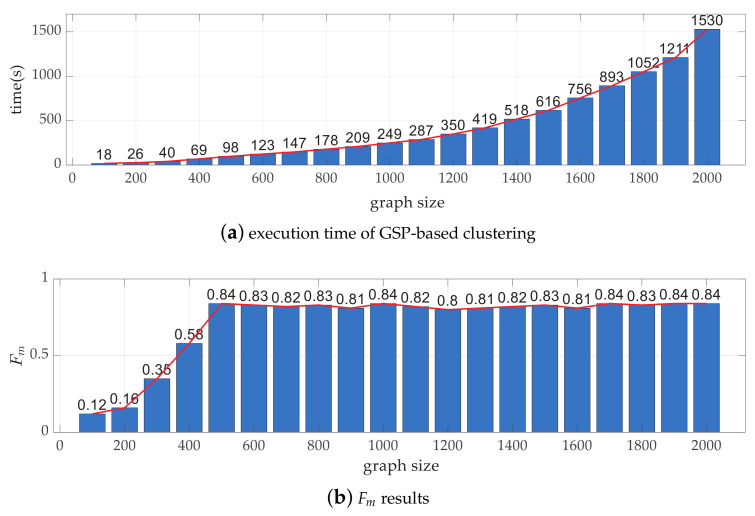
Execution time of GSP-based clustering and $F_{m}$ results for various graph sizes.

Taking the experiment on one-week measurements from AMPds dataset as an example, 500 STSs can be extracted within 2 s. Then, for mapping all extracted STSs, a graph containing 500 nodes can be build, where clustering is carried out costing 7 s, mainly due to pseudo-inverse matrix calculation. Since the computational time of pseudo-inverse matrix depends on matrix size, the efficiency is analyzed under various graph size settings here. The execution time for setting graph size from 100 to 2000 and the corresponding $F_{m}$ results are demonstrated in Figure 5.

In Figure 5a, the longer the graph size is, the larger the graph Laplacian matrix is, leading to time increase in an exponential rate. However, stable NILM performance when graph size is large enough can be observed from Figure 5b.

It takes around 4 ms to extract an STS under our experimental settings shown in Section 4.2. Besides, only 500-byte memory is required for calculating and storing an STS. That is, for typical one-week data, 500 STSs can be extracted and stored in around 250 kb memory. However, a 500 × 500 matrix in GSP requires more than 10 mb memory. Thus, we prefer implementing STS extraction algorithm on edge devices for requiring less transmission and memory resources. Further STS clustering and matching process can be carried out on the cloud for larger capability and faster matrix computation.

### 5.2. NILM Results on REFIT Dataset

**Figure 6:**
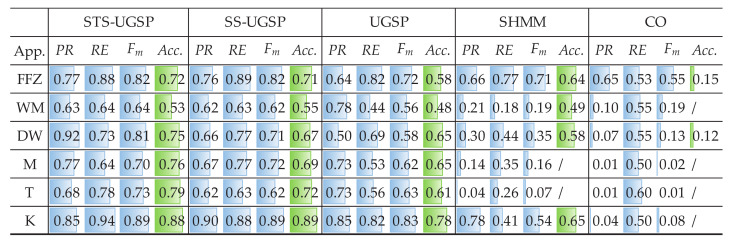
NILM Performance comparison for REFIT House 2.

Since REFIT dataset contains 20 houses, two typical houses are picked for validation, as House 2 and House 6. Both $F_{m}$ and Acc. results for House 2 are shown in Figure 6.

The results shown in Figure 6 is similar to those for AMPds dataset. We can observe that it’s hard for the benchmarks based SHMM and CO to correctly disaggregate any target appliance in House 2. Such inferior results can be explained as numerous unknown loads rise the probability of simultaneous operation of multiple appliances, thus, assigning such states to each appliance becomes more complex. For GSP-based NILM approaches, the results are close to those for AMPds dataset before, i.e., 0.82, 0.78 and 0.69 on overall $F_{m}$, and 0.75, 0.71 and 0.63 on overall Acc., for STS-UGSP, SS-UGSP and UGSP, respectively. Therefore, STS-UGSP succeeds in NILM performance improvement.

It’s worth mentioning that DW and WM operate at similar power ranges, around 2200 W and 2250 W, respectively. Thus, the separation task for DW and WM is beyond the performance boundaries of UGSP. However, DW and WM differ in operation duration, namely, the length of steady-state sequences featured in SS-UGSP, leading to better performance of SS-UGSP against UGSP. Furthermore, the period that DW requires to reach a stable power range after being switched ON is longer than that of WM. Therefore, calculating DTW distance between ‘rising’ STSs in STS-UGSP contributes to distinguishing DW and WM, which leads to disaggregation results refinement. Similar Acc. results are obtained from Figure 6 for most appliances, except WM. The low Acc. results for WM achieved by all NILM methods are due to mismatching with frequent operational cycles of unknown appliances with close power. However, since its power range is almost constant, steady-state power sequences featured in SS-UGSP help improve NILM performance. Benefited from pre-processing, SS-UGSP achieves slightly higher Acc. for K than STS-UGSP.

**Figure 7:**
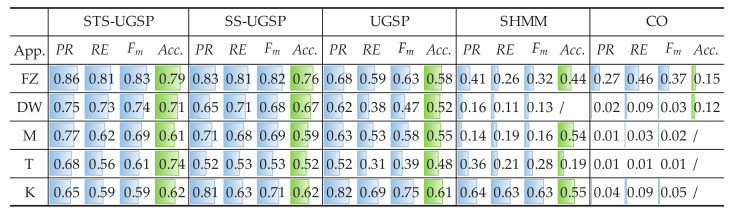
NILM Performance comparison for REFIT House 6.

The experimental results for House 6 from REFIT dataset are demonstrated in Figure 7. Since House 6 has more unknown loads than House 2, NILM performance of all methods drops. In House 6, $F_{m}$ results of T are the lowest in all appliances for SS-UGSP and UGSP. Note that the operational power of T is near 1100 W, close to the peak of FZ loads, above 1000 W while the motor starting to work. Besides, unknown loads with close power ranges to T also exist. Therefore, STS-UGSP is superior to the other methods as state transition details on the power signal are utilized for identification loads.

**Figure 8:**
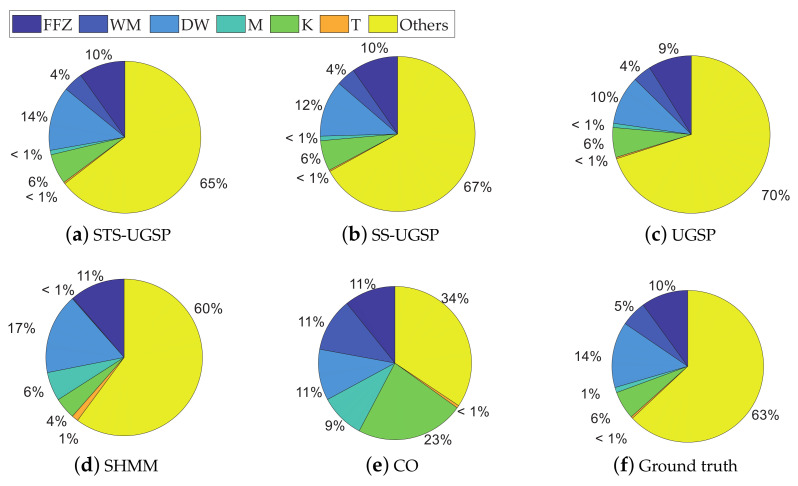
Pie charts of disaggregated energy shares for REFIT House 2.

**Figure 9:**
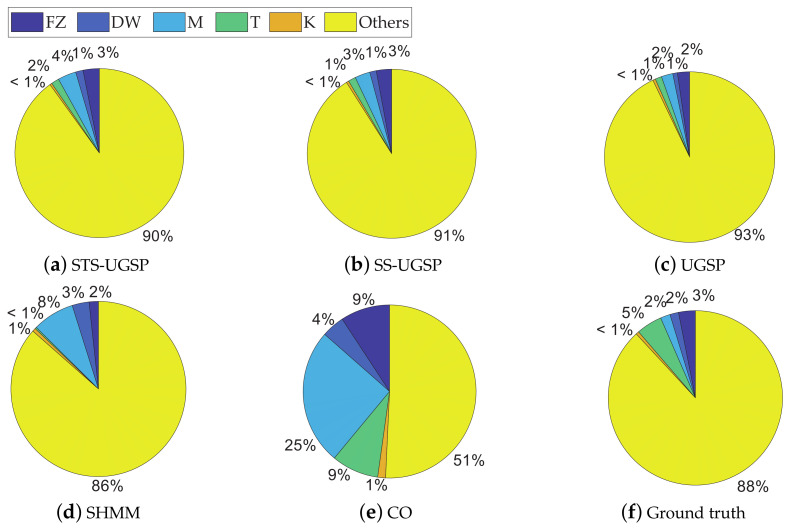
Pie charts of disaggregated energy shares for REFIT House 6.

The energy disaggregated from total power consumption for both House 2 and House 6 are illustrated in Figure 8 and Figure 9, respectively. As mentioned before, target loads account for only a small share of total energy consumption, while other loads account for 63% and 88%, respectively. Thus, disaggregation tasks for such two houses are more difficult than that on AMPds dataset. Comparing to CO, GSP is less sensitive when being applied to such complex NILM tasks. To be specific, over-estimation is observable for CO. e.g., K in House 2 and FZ in House 6 are both over-estimated due to misidentification of other magnitude-wise similar loads. Although SHMM shows more robustness to noisy settings than CO, it performs poor for non-periodic loads like M in both House 2 and House 6.

### 5.3. NILM Results on REDD Dataset

**Figure 10:**
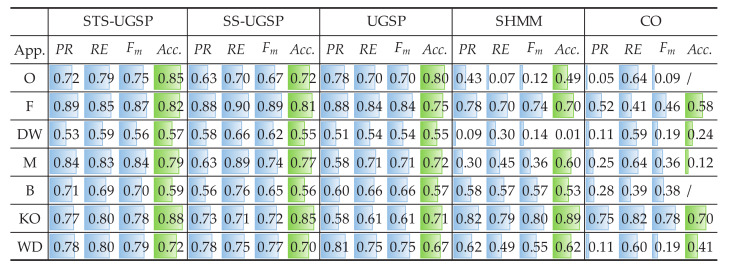
NILM Performance comparison on REDD House 1.

The $F_{m}$ and Acc. results of STS-UGSP and benchmarking methods for REDD House 1 are presented in Figure 10.

**Figure 11:**
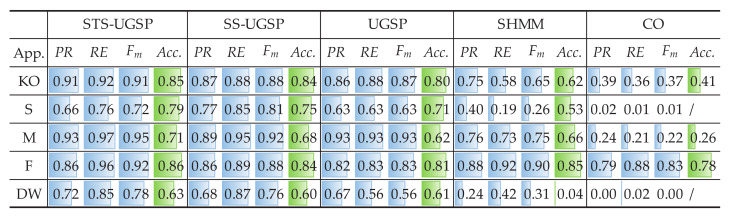
NILM Performance comparison for REDD House 2.

Since REDD House 1 has fewer multi-state loads than in REFIT houses, the overall $F_{m}$ results reach 0.85 for STS-UGSP, 0.78 for UGSP, 0.81 for SS-UGSP, 0.65 for SHMM and 0.48 for CO. For the only multi-state appliance DW (around 200 W, 400 W, 1100 W), worse results comparing to those for other appliances are as expected. The close power ranges of B and M have been claimed in [31], in line with the obtained results for B is affected. The results for REDD House 2 are shown in Figure 11, which are close to the above.

**Figure 12:**
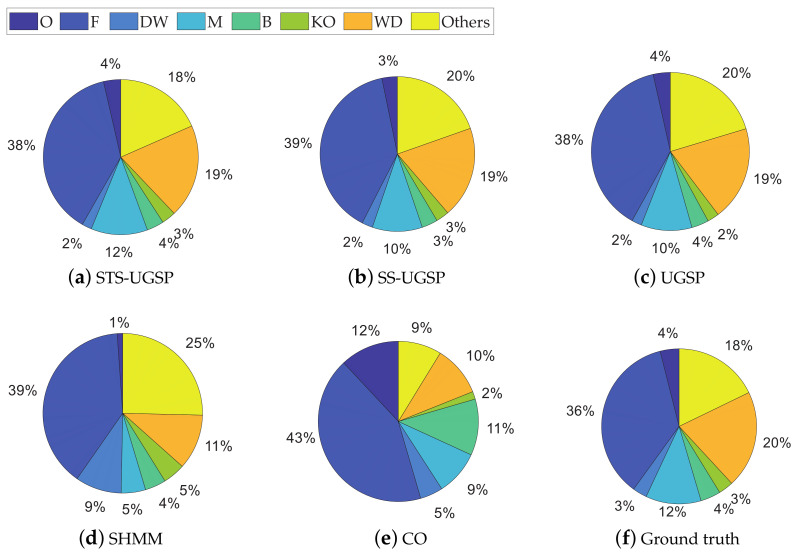
Pie charts of disaggregated energy shares for REDD House 1.

**Figure 13:**
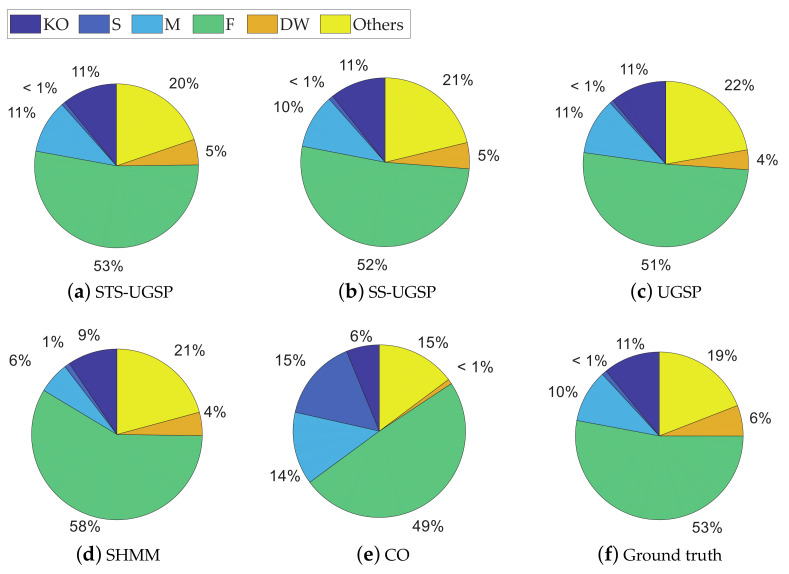
Pie charts of disaggregated energy shares for REDD House 2.

Note that SHMM performs competitively against the others in disaggregating KO in REDD House 1 and F in REDD House 2, while the explanations differ. The active working power of KO is higher than the others in House 1, thus overlapping with low-power loads brings limited impact on disaggregation results. Although F has low working power ranges, its operational cycles are periodic. Besides, the proportions of unknown loads for REDD houses are less than those in the other two datasets, leading to a reduction in misidentification of F, as illustrated in Figure 12 and Figure 13. 

### 5.4. Noise Measurement for All Experimental Cases

**Figure 14:**
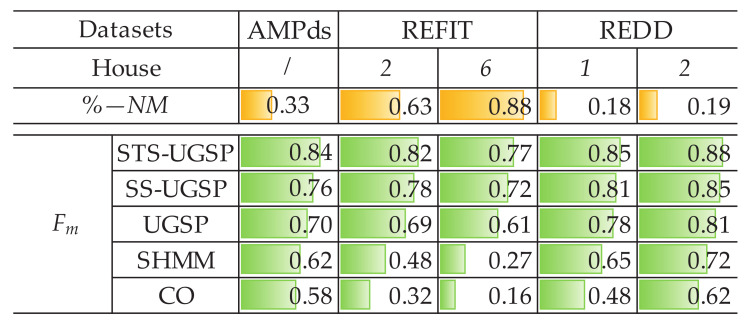
%−NM for various cases and their overall $F_{m}$ across experimental methods.

%−NM is calculated for all cases and shown in Figure 14, with corresponding overall $F_{m}$ results of all methods.

From Figure 14, higher %−NM results for REFIT houses reflect more noises comparing to AMPds dataset and two REDD houses. Higher unknown load shares make disaggregation tasks for REFIT houses more challenging. Therefore, for each NILM approach, its overall $F_{m}$ performance for REFIT houses is generally worse. However, SHMM and CO are more sensitive to unknown loads, leading to sharper drops in $F_{m}$ results.

We can conclude that both SHMM and CO are more sensitive to measurement noise than GSP-based approaches. Since UGSP features power variation, separating loads with close power ranges and identifying loads with long-term power transients become hard. Although SS-UGSP benefits from the steady-state sequence feature, outperforming UGSP in disaggregation for most appliances, it performs poor for short-lasting loads. With respect to STS-UGSP, its better NILM performance against others in various metrics is benefited from the proposed STS feature with a forward-backward matching process.

## 6. Conclusions

In this paper, an unsupervised GSP-based approach to disaggregate loads on low-rate power measurements is proposed. Driven by the features utilized in the existing GSP-based methods carry limited information thus limiting NILM performance, in the proposed STS-UGSP, power STSs containing complete operational state transients are extracted for calculating DTW distances. By mapping such STSs to graph nodes and weighting edges based on their DTW distances, they can be grouped into various clusters. Then, a forward-backward STS matching algorithm is proposed to search optimal STS pairs based on power and temporal information. Experiments are carried out on open-access AMPds, REFIT and REDD datasets. The results show GSP performs more robust than classic SHMM and CO in the scenarios with high proportion of unknown loads. Besides, STS features and their further matching help improve NILM performance, especially for the loads with a spike in power signal due to motor activation and those with turn-ON transients longer than the sampling period. Future work includes refining STS matching via further mining temporal connection among state transitions of multi-state loads; adding matching between STSs and steady-state power sequences for improving power signal reconstruction; utilizing DTW distance to calculate magnitude similarity between STSs when matching and applying the proposed method on the measurements with various low frequencies.

## Figures and Tables

**Table 1:** Parameter settings.

Parameters	T	$t_{0}$	$t_{1}$	τ	γ	α	β	ρ	$j_{0}$	*q*
AMPds	50	1	1	500	0.2	0.5	0.5	0.01	50	0.5
REFIT	50	2	2	1000	0.2	0.9	0.1	0.01	50	0.5
REDD	50	3	3	2000	0.2	0.5	0.5	0.01	50	0.5

## Data Availability

Not applicable.
